# Insights into the Bacterial Profiles and Resistome Structures Following the Severe 2018 Flood in Kerala, South India

**DOI:** 10.3390/microorganisms7100474

**Published:** 2019-10-19

**Authors:** Soumya Jaya Divakaran, Jamiema Sara Philip, Padma Chereddy, Sai Ravi Chandra Nori, Akshay Jaya Ganesh, Jiffy John, Shijulal Nelson-Sathi

**Affiliations:** Interdisciplinary Biology, Rajiv Gandhi Centre for Biotechnology (RGCB), Thiruvananthapuram 695 014, Kerala, India; soumyajd@rgcb.res.in (S.J.D.); jamiemasara@rgcb.res.in (J.S.P.); chereddypadma@rgcb.res.in (P.C.); ravichandranori@outlook.com (S.R.C.N.); akshayjganesh17@iisertvm.ac.in (A.J.G.); jiffy@rgcb.res.in (J.J.)

**Keywords:** metagenomics, flood, antibiotic resistance, bacterial diversity, resistome

## Abstract

Extreme flooding is one of the major risk factors for human health, and it can significantly influence the microbial communities and enhance the mobility of infectious disease agents within the affected areas. The flood crisis in 2018 was one of the severe natural calamities recorded in the southern state of India (Kerala) that significantly affected its economy and ecological habitat. We utilized a combination of shotgun metagenomics and bioinformatics approaches to understand the bacterial profile and the abundance of pathogenic and antibiotic-resistant bacteria in extremely flooded areas of Kuttanad, Kerala (4–10 feet below sea level). Here we report the bacterial profiles of flooded sites that are abundant with virulent and resistant bacteria. The flooded sites were heavily contaminated with faecal contamination indicators such as *Escherichia coli* and *Enterococcus faecalis* and multidrug-resistant strains of *Pseudomonas aeruginosa, Salmonella typhi/typhimurium, Klebsiella pneumoniae, Vibrio cholerae*. The resistome of the flooded sites contains 103 known resistant genes, of which 38% are plasmid-encoded, where most of them are known to be associated with pathogenic bacteria. Our results reveal an overall picture of the bacterial profile and resistome of sites following a devastating flood event, which might increase the levels of pathogens and its associated risks.

## 1. Introduction

Flooding is one of the most destructive natural disasters, resulting in significant damages to life and infrastructure worldwide [1]. The devastating flooding in the southern state of India (Kerala) during August 2018 was declared as a “calamity of severe nature”, leaving 23 million people affected [2,3]. This was the worst ever flood in the history of Kerala since the Great flood in 1924 [4]. During this season, Kerala state received a cumulative rainfall of 2346.3 mm, 42% greater than the monsoon average [5]. 35 out of 54 dams within the state were opened due to the heavy rainfall in its catchment areas. The flood left 10,319 houses fully damaged, more than 0.1 million houses partially damaged, destroyed 83,000 km of roads, including 10,000 km of major roads, and 60,000 hectares of crops causing nearly $2.9 billion worth of damage [2,3]. Previous epidemiological evidence suggests that floods are positively associated with increased risk of water-borne and vector-borne diseases such as skin infection, typhoid fever, cholera, leptospirosis, hepatitis A, malaria, dengue fever, yellow fever and West Nile fever [6,7]. The stagnant floodwater can also significantly affect the environmental microbiome and spread of microbial pathogens [8].

Furthermore, there are a number of previous reports available on the impact of the raw sewage from domestic waste, livestock, hospitals [9], industries [10], agriculture lands, and wastewater treatment plants [11] in the dissemination of pathogenic bacteria and antibiotic resistance genes in the natural environment [12]. The untreated sewage and effluents from wastewater treatment plants [13] discharged into lakes, rivers, [14] and sea made them as putative reservoirs for antibiotic-resistant bacteria and genes [14,15,16]. During severe flooding, this contaminated water from rivers, sea, as well as causing runoff from urban, clinical, agricultural and livestock conglomerates into the natural environment. This facilitates the spreading of antibiotic-resistant bacteria and resistant genes among the bacterial population [16] by Horizontal Gene Transfer (HGT) through plasmid, transposons, and integrons [17].

Previous studies showed floods due to Hurricane Katrina in the US, 2005 [18], Chennai flood in 2015 [19], Hurricane Harvey in Houston, 2017 [20], and Thailand flood in 2011 [21], significantly influenced the bacterial profile of water and soil. Reports on these floods evidenced that faecal contamination indicators like *Escherichia coli*, *Enterobacter aerogenes* and *Enterococcus* were widely distributed in water and soil sediments. In addition, infectious disease-causing pathogens such as *Legionella pneumophila*, *Vibrio cholerae, Aeromonas hydrophila*, *Klebsiella pneumoniae, Clostridium perfringens, Salmonella typhi*, *Streptococcus pyrogens* and *Shigella flexneri* were also abundant at flooded sites [20]. Another important concern is the possible coexistence of multidrug-resistant pathogenic bacteria with environmental bacteria, especially since the frequency of flooding could increase in the coming years due to global climate change and expansion of coastal cities [22]. A previous study at Hurricane Harvey’s flooded sites in Houston revealed the elevated levels of anthropogenic antibiotic-resistant markers such as *sul1* and *intI1* [20]. There were attempts to understand flood associated microbial composition alteration [8,18,20] and circulation of pathogenic and resistant bacteria, but its influence varies significantly based on the geography and nature of flooded environments. Due to the unpredictable nature of floods, there is usually an absence of pre-flood data, which also makes such studies more challenging [23].

A better understanding of the impact of extreme flooding on the disruption of natural environmental microbiome and dissemination of pathogenic and antibiotic-resistant bacteria still needs detailed investigation. Kuttanad, an agricultural region in Kerala located in India’s lowest altitude of 4–10 feet below sea level, is particularly susceptible to flood damage due to its unique geography [24]. Here, we employed shotgun metagenomic and bioinformatics techniques to understand the bacterial community profile and the resistome of flood-affected areas of Kuttanad, India. We found a wide range of bacterial communities and a higher abundance of multidrug-resistant pathogenic species in flood-affected areas. Our results will provide a better understanding of the bacterial profile of extremely flooded settings and provide more evidence to support decision-making for the prevention and control of flood-related disease outbreaks.

## 2. Results

### 2.1. Bacterial Profiles of Extremely Flooded Sites

**Figure 1:**
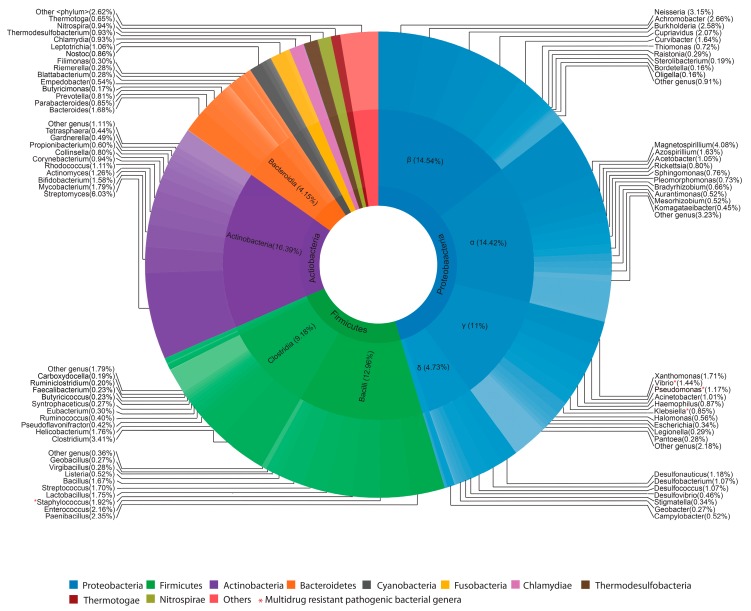
Bacterial taxonomic distribution of flooded sites. Sunburst plot showing the taxonomic classification and relative abundance of bacterial species in flooded sites. The taxonomic phylum is represented in the innermost ring, class in the middle, and genus are represented in the outermost ring of the circle. Within each taxonomic classification, taxa are sorted according to its abundance. A red * symbol represents the multidrug-resistant pathogenic bacteria present in flooded sites. See Table S1 for the full list of bacterial taxa found in flooded sites.

In total, 178,527 16S rRNA reads were obtained from sediment samples collected from five different sites that were severely affected by the flood in August 2018. Only less than half of the 16S rRNA reads (48%) were able to taxonomically classify into known microorganisms, of which the majority (96%) belongs to bacterial species. Among the annotated cases of bacterial species, Proteobacteria (45.4%) was found to be the most abundant phylum followed by Firmicutes (23%), Actinobacteria (16.39%) and Bacteriodetes (5.94%) (Figure 1, Table S1). Within the Proteobacteria phylum, the most abundant classes were the species of *Betaproteobacteria* (14.54%) and *Alphaproteobacteria* (14.42%) followed by *Gammaproteobacteria* (11%) and *Deltaproteobacteria* (4.73%). Within Firmicutes, the most abundant species were from *Bacilli* (12.96%) followed by *Clostridium* (9.18%) class. *Actinobacteria* (16.39%) was found to be the dominant class in the phylum Actinobacteria. The Bacteriodetes primarily consisted of *Bacteriodia* (4.15%) and *Flavobacteria* (1.27%). At the genus level, *Streptomyces* (6.03%) is the abundant genera followed by *Magnetospirillum* (4.08%), *Neisseria* (3.15%) and *Achromobacter* (2.66%). The bacterial community richness and diversity (chao1, Shannon indices) of flooded sites were found to be uniform (Table S2).

In addition, we also found many virulence factors (VFs) in flooded sites that are distributed among the bacterial pathogens such as *Pseudomonas aeruginosa, Escherichia coli, Acinetobacter baumannii, Klebsiella pneumoniae, Salmonella enterica, Vibrio cholerae, Enterococcus faecalis,* and *Staphylococcus aureus.* The functional classification of these VFs showed that they are involved in bacterial motility, cell adherence, iron uptake, secretions and toxins (Table S3). These VFs are associated with pathogenic mechanisms in clinically relevant bacteria.

### 2.2. Resistome of Flooded Sites

**Figure 2:**
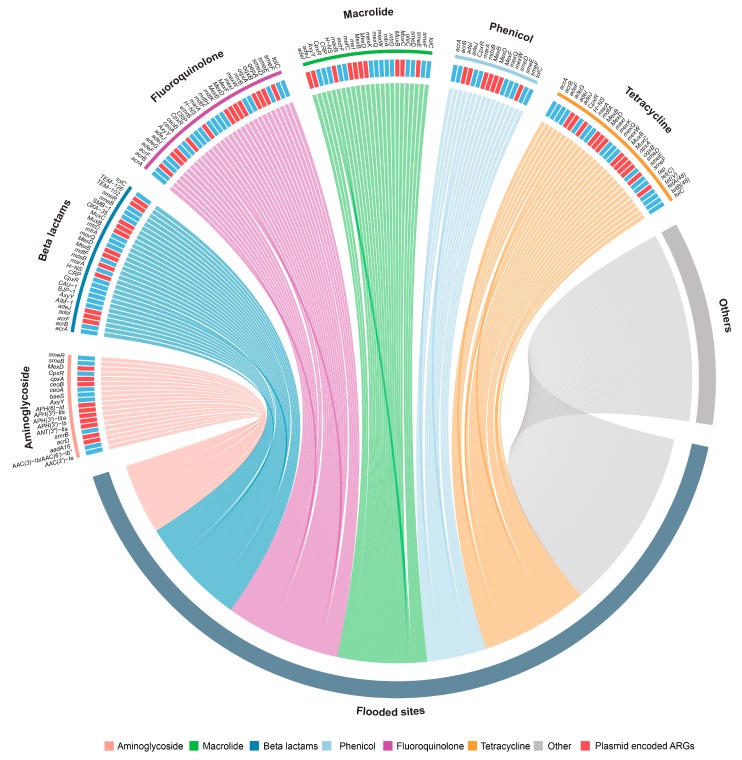
Resistome of the flooded sites. Chord diagram showing the presence of Antibiotic Resistance Genes (ARGs) detected in flooded sites. ARGs in flooded sites were classified into 6 major drug classes. The coloured edges represent the proportion of ARGs of different drug classes detected in flooded sites. ARGs conferring resistance to aminocoumarin, sulfonamide, mupirocin, rifampicin, triclosan, glycopeptide and diaminopyrimidine classes of antibiotics are represented as others category. Red blocks indicate plasmid-encoded ARGs. A complete list of antibiotic resistance genes and their characteristics are listed in Table S4.

To test the prevalence of Antibiotic Resistance Genes (ARGs) in the flooded sites, resistome profiles were reconstructed from the sediment samples collected from five different flooded sites. In total, the resistome of flooded sites contains 103 unique genes that confer resistance to antibiotics over 12 different classes (Figure 2, Table S4). Relatively similar gene distribution was present with an average number of 46 ARGs in each flooded site. Among the major resistance classes, most of the ARGs present in flooded sites confer resistance to aminoglycoside (19 genes), beta-lactams (29 genes), tetracycline (29 genes), fluoroquinolone (31 genes), macrolide (24 genes) and phenicol (16 genes). 38% of the detected ARGs in flooded sites were multidrug-resistant, the most frequent being *MexB*, *MexF* and *MuxB*. These genes are known to be encoded in plasmids and confer resistance against beta-lactams, fluoroquinolones, macrolides and phenicols.

Carbapenems, a subfamily of beta-lactam antibiotics, currently are the most effective broad-spectrum antibiotics [25], for which 10 resistant genes (eg., *mdsB*, *MexB, mexQ*) were found in the flooded sites. Additionally, we also detected genes that confer resistance to synthetic antibiotics such as sulfonamide (*sul1, sul2 and sul4*), fluoroquinolones (e.g., *smeE, adeF, acrB*) and penems (*TEM-126* and *TEM-102*). Furthermore, most of these genes were predominantly reported in species such as *Pseudomonas aeruginosa, Escherichia coli, Acinetobacter baumannii, Klebsiella pneumoniae, Shigella flexneri, Vibrio cholerae, Enterococcus faecalis, and Staphylococcus aureus* (Table S4). There are about 39 (38%) resistant genes that are plasmid-encoded, which increase the chances of conjugative transfer than non-ARG carrying plasmids.

### 2.3. Viability of Pathogenic and Resistant Bacteria in Flooded Sites

Based on the colour and morphology of bacterial colonies in selective media, we confirmed the presence of faecal contamination indicators such as *Enterococcus faecalis, Escherichia coli* and pathogenic bacterial species such as *Staphylococcus aureus, Salmonella typhi/typhimurium, Pseudomonas aeruginosa, Vibrio cholerae* and *Klebsiella pneumoniae.* The abundance of these bacterial species was calculated by colony-forming unit(CFU)/gram of dry weight. The faecal contamination indicating bacteria such as *Enterococcus faecalis* (8.4 ± 0.5 × 10^3^ CFU/gram of dry weight) and *Escherichia coli* (3 ± 0.35 × 10^3^ CFU/gram of dry weight) showed 2- to 6-fold higher abundance than comparable settings [26] (Table S5). *Staphylococcus aureus*, an opportunistic pathogen, was the most abundant bacteria (8.5 ± 3 × 10^5^ CFU/gram of dry weight) followed by *Salmonella typhi/typhimurium*, *Vibrio cholerae*, *Klebsiella pneuomiae* and *Pseudomonas aeruginosa*.

**Figure 3:**
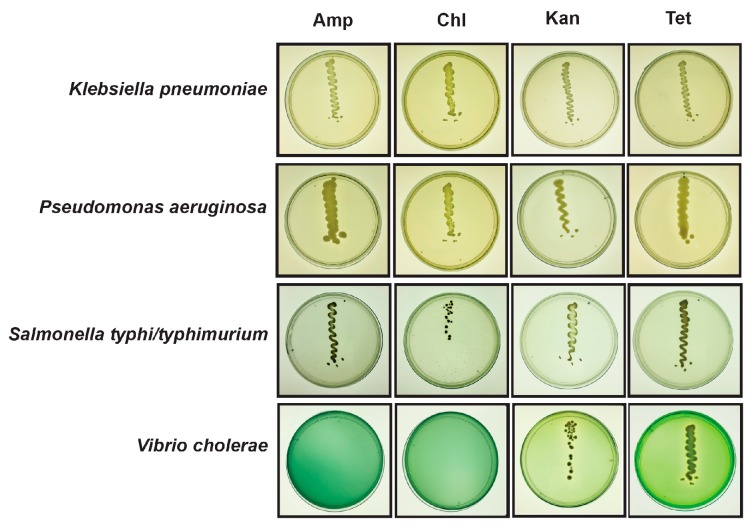
In vitro evaluation of antimicrobial resistance of pathogenic bacterial species isolated from flooded sites (August 2018). The culture plates showing pathogenic bacteria (*Klebsiella pneumoniae, Pseudomonas aeruginosa, Salmonella* *typhi/typhimurium*, *Vibrio cholerae)* streaked on selective/differential agar media (HiCrome^TM^ Klebsiella selective agar base, Cetrimide agar base, Wilson Blair agar with brilliant green (w/BG), Thiosulphate-Citrate-Bile-Salt sucrose (TCBS) agar, respectively) containing different antibiotics such as Amp: Ampicillin (100 µg/mL), Kan: Kanamycin (50 µg/mL), Chl: Chloramphenicol (25 µg/mL), Tet: Tetracycline (10 µg/mL), and incubated at 37 °C for 1–3 days.

The 24 morphologically distinct colonies of these six pathogenic species identified in flooded sites were subjected to further antimicrobial susceptibility analysis against four different antibiotics such as ampicillin (100 µg/mL), chloramphenicol (25 µg/mL), kanamycin (50 µg/mL), and tetracycline (10 µg/mL). These antibiotics belong to major classes such as beta-lactam, aminoglycoside, phenicol and tetracycline, respectively. Among these 24 isolates, 7 isolates were multidrug-resistant, and species identity was confirmed by 16S rRNA sequencing using universal primers. Interestingly, these isolates belong to *Pseudomonas aeruginosa*, *Salmonella typhi/typhimurium*, *Klebsiella pneumoniae* and *Vibrio cholerae* (Figure 3). Additionally, the faecal contamination indicator *Escherichia coli* showed resistance against ampicillin, and *Staphylococcus aureus* was sensitive to all antibiotics tested.

## 3. Materials and Methods

### 3.1. Sample Collection

**Figure 4:**
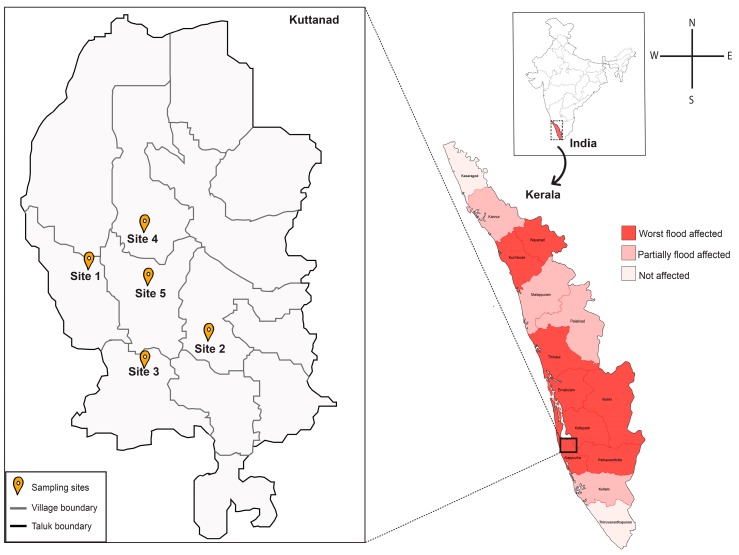
Map showing flooded regions and sampling sites of state Kerala, India. The intensity of the red colour indicates the level of flood severity in fourteen districts of Kerala during August 2018. Triplicate samples were collected from each site during August 2018.

To investigate the bacterial profile of extremely flooded sites, we performed a detailed metagenomic screening of sediment samples collected from Kuttanad, the lowest altitude in India (4–10 feet below sea level) [24] which covers over 500 sq km (Figure 4). Soil/sediment samples were collected from five sites of Kuttanad namely Nedumudi (9°26′33.73″ N, 76°24′26.565″ E), Ramankary (9°24′47.808″ N, 76°27′19.152″ E), Thakazhy (9°23′5.352″ N, 76°26′42.971″ E), Pulinkunnu (9°26′55.464″ N, 76°26′47.76″ E), Mankombu (9°25′19.056″ N, 76°28′19.92″ E) during the flood season (August 2018). As sampling sites were public places, special permits were not required for sample collection. Triplicate sediment samples were collected from each place in sterile 50 mL conical tubes by using sterile steel scoops, and unique identifiers were given for each sample. Following sample collection, 50 mL conical tubes were wrapped with parafilm and transported to the laboratory on ice (4 °C). The pH for soil/sediment samples was measured with pH meter in a suspension of a 1:5 ratio of soil to ultrapure water on the day of sampling. The sediment samples were stored in the deep freezer at −80 °C and −20 °C for DNA extraction and cultured based analysis, respectively. Geographical location, environmental indices and pH at the time of sampling were recorded (Table S6).

### 3.2. DNA Extraction

Metagenomic DNA from sediment samples was extracted using DNeasy^®^ Power Soil^®^ kit (Qiagen, Germany), and slight modifications were made in the metagenomic procedure such as the addition of ribonuclease A (RNase A; 1 µg/mL, Qiagen, Germany) along with solution C2 (inhibitor removal solution). 1 h incubation at 37 °C, and two additional washes were performed with 70% ethanol [27]. The purity and concentration of the metagenomic DNA was measured using a multimode microplate reader (TecanSpark 10m, Tecan, Switzerland). The integrity of extracted DNA was confirmed by agarose gel electrophoresis by loading an equal amount of extracted DNA on agarose gel along with גHindIII digest marker (New England Biolabs, Ipswich, MA, USA) [28]. An equal amount of DNA isolated from triplicate samples of each site was pooled and used for shotgun metagenomic sequencing.

### 3.3. Shotgun Metagenomic Sequencing and Analysis

One microgram of high-quality metagenomic DNA was further used for shotgun metagenomic sequencing and libraries were prepared using the NEBNext Ultra DNA Library preparation kit following the manufacturer’s protocol. In brief, the DNA is subjected to a sequence of enzymatic steps for repairing the ends and tailing with dA-tail followed by ligation of adapter sequences. These adapter-ligated fragments were then cleaned up using Solid Phase Reversible Immobilization (SPRI) beads. The cleaned fragments were indexed using the polymerase chain reaction (PCR) cycle to generate final libraries for paired-end sequencing. 2 × 151 bp sequencing reads were generated on the Illumina HiSeq system, yielding about 3 GB of data per sample.

The quality of paired-end sequences was assessed using FastQC v0.11.4 [29]. Reads with low quality (Q value 20 cutoff) and adapter sequences were trimmed and removed using FastX toolkit v.0.0.13.2 [30]. SortMeRNA [31] was applied to the filtered shotgun metagenome data to extract 16S rRNA sequences from filtered reads. For each 16S rRNA sequence, a BLASTx search was performed against the NCBI non-redundant protein database using DIAMOND v.0.9.24 [32]. Output data were analyzed with the MEtaGenome ANalyzer (MEGAN v5.7.1) [33], using the following settings: Min Score = 50, top Percent = 10, min Support = 1, min-complexity filter = 0. Comparative analysis for taxa in terms of percentage mean relative frequency was performed using Statistical Analysis of Metagenomic Profiles (STAMP; V2.1.3) [34], G-test (with Yates’) + Fisher’s was applied to compare bacterial communities pairwise (flooded and natural ecosystems) with *p*-value <0.05, confidence intervals of 95%, and extended error bars were plotted. Genus-level taxa abundances were used in STAMP [34] to generate principal component analyses (PCA). Alpha diversities were measured by the Shannon diversity index and chao1 index using Qiime2 [35] to analyze the diversity within the samples.

Antibiotic resistance and virulence genes were identified by mapping the filtered reads against Comprehensive Antibiotic Resistance Database (CARD) [36], and the Virulence Factor Database (VFdb) [37] using DIAMOND v.0.9.24 (BLASTx, -e 1e-05). Only hits with sequence identity above 90% and an alignment length over 25 amino acids were kept [38,39]. The identified ARGs were further annotated according to the corresponding CARD database descriptions. The occurrence of ARGs in plasmids were determined using BLASTn [40] against 14,595 complete plasmid sequences from the NCBI RefSeq database (updated on 16 May 2019). Hits with >70% of query coverage and >70% identity were kept.

Publicly available metagenomic datasets collected from local mangrove ecosystems that lie across the state of Kerala reported by Imchen et al. [41] was used for comparative analysis. The metagenomes with the following accession numbers were downloaded from the Sequence Read Archive (SRA) database: SRR2844600, SRR2844601, SRR2844602, SRR2844616.

### 3.4. Abundance of Selected Pathogens

The abundance of selected pathogenic bacteria was calculated by CFU. One gram of sediment sample was pooled from triplicate samples of five flooded sites and mixed with 9 mL sterile phosphate-buffered saline taken in test tubes. The solution was homogenized using a vortex machine, and the suspension was serially diluted up to 10^-4^ [42,43]. 100µL of diluted suspension from each dilution was spread on different selective media such as Eosin Methylene Blue agar (EMB Agar, HiMedia, India), Salmonella differential agar modified (HiMedia), HiCrome^TM^ Klebsiella selective agar base (HiMedia), Mannitol salt agar (HiMedia), Thiosulphate-Citrate-Bile-Salt-sucrose agar (TCBS agar, HiMedia), Cetrimide agar base (HiMedia), Wilson-Blair agar with brilliant green (WB agar w/BG, HiMedia), and Enterococcus differential agar base (HiMedia), by using a sterilized glass spreader and incubated at 37 °C for 1–3 days, depending upon the bacteria that were grown in the selective media. Control plates were spread with only the phosphate-buffered saline and processed in parallel with soil suspension as method blank. After the incubation period, the number of colonies from each plate was counted, and CFU of wet soil was calculated by the following Equation (1):

CFU/gram = Number of colonies/(dilution plated × dilution factor) (1)

Colony-forming unit of soil bacteria is expressed in dry weight instead of one gram of wet soil. Thus, one gram of sediment samples were pooled from flooded sites and then air-dried to determine the percentage change. Percentage change is calculated by the following Equation (2):

[(wet weight − dry weight)/wet weight] * 100 = % change(2)

This percentage change value is used to convert CFU per gram wet soil to CFU per gram dry soil. To minimize error, all these experiments were repeated thrice for the sediment samples collected during the flood and the result is expressed in CFU/gram of dry weight (mean ± s.d).

### 3.5. Identification of Antibiotic-Resistant Bacterial Pathogens

Bacterial pathogens that grow abundantly and show characteristic colony morphology such as (i) purple-magenta coloured or cream to white coloured colonies on HiCrome^TM^ Klebsiella selective agar base; (ii) yellowish-green colonies on Cetrimide agar base; (iii) black with sheen colonies on Wilson Blair agar with BG; (iv) round yellow colonies on TCBS agar; (v) yellow or white colonies surrounded by a yellow zone on Mannitol salt agar; and (vi) purple with black centre and green metallic sheen colonies in EMB agar were used to check resistance against ampicillin (Sigma-Aldrich, St.louis, MO, USA), chloramphenicol (Sigma-Aldrich), kanamycin (Sigma-Aldrich), and tetracycline (Sigma-Aldrich). Pure colonies were picked from the selective media and incubated in 5 mL nutrient broth for 1–3 days at 37 °C for 200 rpm in Innova shaker (New Brunswick^TM^ Innova^®^ (40/40R)). After incubation, a loop full of bacterial culture was streaked on respective selective media containing antibiotics such as ampicillin sodium salt (100 µg/mL), chloramphenicol (25 µg/mL), kanamycin sulfate (50 µg/mL), and tetracycline hydrochloride (10 µg/mL), respectively. The plates were incubated at 37 °C for 1–3 days according to the bacteria that streaked on the media. The bacterial isolates, which show resistance to more than one class of antibiotics, were considered as multidrug-resistant [44]. A loop full of nutrient broth streaked on the antibiotic-containing media is used as a method blank and is processed parallel with bacterial culture.

### 3.6. 16S rRNA Sequencing

Bacteria showing antibiotic resistance were picked from the selective media and incubated in 10 mL Luria Bertani (LB) broth for 12 h. After incubation, bacterial genomic DNA was isolated using the Nucleospin^®^ Microbial DNA Kit (Macherey Nagel, Duren, Germany) according to the manufacturer’s protocol. The quality and concentration of genomic DNA was measured by using a multimode microplate reader (Tecan Spark 10 m, Tecan, Switzerland) and high-quality genomic DNA was used for 16S rRNA sequencing. 16S rRNA gene was amplified using a universal forward primer (5′CAGGCCTAACACATGCAAGTC3′) and reverse primer (3′GGGCGGWGTGTACAAGGC5′) [45]. PCR was carried out in a 20 µL reaction volume which contained 1X PCR buffer (100mM Tris HCl, pH-8.3; 500mM KCl), 0.2mM each dNTPs, 2.5mM MgCl_2_, 1 unit of AmpliTaq Gold DNA polymerase enzyme (Applied Biosystems, Foster City, CA, USA), 0.1 mg/mL BSA, 4% DMSO, 5 pM of forward and reverse primers, and template DNA in Gene Amp PCR System 9700, (Applied Biosystems, Foster City, CA, USA). PCR conditions were: 1 cycle of 95 °C for 5 min followed by 35 cycles of 95 °C for 30 s, 65 °C for 40 s, 72 °C for 60 s and 72 °C for 7 min. The PCR product was checked on agarose gel electrophoresis by loading 5 µL of PCR product on to 1.2% (w/v) agarose gel and the gel was visualized using UV transilluminator (Genei, India) and the image was captured using Gel documentation system (Bio-Rad, Berkeley, CA, USA). Amplified products were re-purified by using ExoSap (Thermo Fisher Scientific, Waltham, MA, USA) treatment. Sequencing PCR was carried out by using the Big Dye Terminator v3.1 Cycle Sequencing Kit (Applied Biosystems, Foster City, CA, USA) in a thermal cycler (Gene Amp PCR System 9700, Applied Biosystems) according to the manufacturer’s instructions [46]. The forward and reverse sequences obtained from Sanger sequencing were merged using Bioedit v7.1 [47]. Further, the merged sequences were used for the identification of species using BLASTn search against the reference data sets of NCBI.

### 3.7. Availability of Data

The raw metagenomic data reported in this paper have been deposited in the NCBI SRA database with accession numbers SRR9620086, SRR9620087, SRR9620088, SRR9620089, and SRR9620090, as part of BioProject PRJNA552210.

## 4. Discussion

Extreme flooding is one of the major risk factors for human health. It can significantly alter the top layer soil microbiome of flooded sites, and enhance the mobility of infectious disease agents, especially the water-borne pathogens such as *Salmonella typhi*, *Vibrio cholerae*, *Leptospira sp.* and its resistant strains [48]. Shotgun metagenomics of sediment samples collected from extremely flooded sites revealed the overall bacterial profile and resistome at these settings. However, a large portion (52%) of the bacterial diversity of these flooded sites still remains unknown.

Due to the unpredictable nature of floods, there is no metagenomic data available from the studied settings before flooding for understanding the exact influence of flood on the bacterial communities. A comparison of flooded sites with publicly available metagenomic data [41] of the local mangrove ecosystem showed a significant difference (*p* < 0.05) in bacterial communities between both settings (Figure S1). Interestingly, the PCA analyses showed that bacterial community composition was similar in different flooded sites and it significantly differs from the local mangrove ecosystems (Figure S2). In addition, we also found that the number of ARGs present in flooded sites is three times higher compared to the local mangrove ecosystems and only 27 ARGs (26%) were found to be common between both sites (Figure S3). In order to check the viability and resistance of pathogenic bacteria present in flooded sites, we performed a culture-based analysis using different selective and differential agar media. Most of the microbes detected are environmental bacteria but faecal contamination indicators and clinically relevant pathogens such as *Vibrio cholerae, Klebsiella pneumoniae, Salmonella typhi/typhimurium*, etc., and its resistant strains were also abundant. The higher levels of bacterial contamination and dissemination of resistant pathogenic bacteria at the flooded areas might cause water-borne and vector-borne diseases [49] such as dysentery, cholera, typhoid fevers and other gastrointestinal diseases.

As previously reported by Garner et al. a large number of resistant genes which show resistance to multiple classes of antibiotics were observed in flooded sites in Colorado in 2013 [50]. A similar proportion of multidrug-resistant genes were observed at the flooded sites and after further annotation, we identified the efflux pump as the most common mechanism for antibiotic resistance. Further, we found a high prevalence of resistant genes that are associated with clinically relevant bacterial pathogens and their viability was confirmed by culture-based techniques. The coexistence of bacterial species of different drug resistance levels could increase the chance of resistant genes being exchanged between strains of pathogenic and non-pathogenic bacteria [51]. Up to 46% of the multidrug-resistant genes identified were found to be plasmid-encoded, which increases the transfer potential of these genes [51,52]. These genes are having a higher transfer potential, yet we could not precisely quantify the extent to which lateral gene transfer can promote further gene mobility. These results indicate that flooded sites are large reservoirs of antibiotic resistance genes.

Yu et al. reported that elevated levels of faecal contamination indicators such as *Escherichia coli* and *Enterococcus* species, and pathogenic bacteria such as *Pseudomonas aeruginosa* and *Klebsiella pneumoniae* were abundantly present in the flood-affected areas of Houston [20]. Additionally, our results suggest a higher abundance of *Escherichia coli* and *Enterococcus* species in heavily flooded sites of Kuttanad which might be due to the overflow of sewage during the flood. The abundance of faecal indicating bacteria could increase the risk of gastroenteritis, diarrheal diseases and skin infections [53,54,55]. The culture-based analysis clearly indicates the presence of multidrug-resistant *Pseudomonas aeruginosa,* an opportunistic pathogen that comes under the critical priority list of WHO [56] and *Klebsiella pneumoniae*, one of the biggest threats to human health [56]. Another study by Emerson et al. reported that *Pseudomonadaceae* and *Enterobacteriaceae* are the most abundant taxa in flooded homes in Colorado, USA [57]. Other important antibiotic-resistant pathogenic bacterial species such as *Vibrio cholerae,* causative agent of cholera [58], and *Salmonella typhi/typhimurium,* responsible for gastroenteritis, were present in heavily flood-affected areas of Kuttanad. In addition, similar bacterial species were reported in water samples collected after the flood in Chennai, India [19]. Interestingly, the multidrug-resistant pathogenic species such as *Klebsiella pneumoniae*, *Pseudomonas aeruginosa*, *Salmonella typhi/typhimurium* and *Vibrio cholerae* were found in post-flood samples collected after six months of the flood event in February 2019. This indicates the persistence of pathogenic and resistant bacterial species even after the devastating flood. Furthermore, better time-resolved sampling is required to estimate pathogen survival duration, the source of detected pathogens and resistant strains, and their evolution [20].

## 5. Conclusions

Our results indicate that the devastating flood that occurred in the southern state of India might have influenced the bacterial composition of its watershed areas. The higher abundance of multidrug-resistant pathogens such as *Klebsiella pneumoniae*, *Pseudomonas aeruginosa*, *Salmonella typhi/typhimurium* and *Vibrio cholerae* is alarming because it could make post-flood disease outbreaks difficult to treat. Our study provides better insights into the pathogenic and resistance traits of bacterial communities in flooded sites, which helps to plan better preventive measures against post-flood disease outbreaks, which include preventive measures such as chlorination of floodwater, vaccination, and good hygienic practices that can help avoid the spread of infectious diseases.

## Supplementary Materials

The following are available online at https://www.mdpi.com/2076-2607/7/10/474/s1. Figure S1: Distribution of 77 genera found at significantly different abundances in the metagenomics profiles of flooded and local mangrove settings as identified by STAMP analysis; Figure S2: Principal component analysis (PCA) based on relative abundance of bacterial taxa at genus level in local mangrove (blue) and flooded (orange) sites using Welch’s t-test two sided (*p* < 0.05); Figure S3: Chord diagram showing the presence of Antibiotic Resistance Genes (ARG) detected in flooded (green) and local mangrove (grey) settings; Table S1: Table showing the relative abundance of bacterial communities in flood-affected areas across phylum, class and genus levels; Table S2: Table showing the biodiversity indices of bacterial communities in flooded sites; Table S3: Functional annotation of the virulence factors distributed in pathogenic species found in the flooded sites; Table S4: List of ARGs found in flooded and local mangrove settings with its drug class, resistance mechanism, presence of mobile genetic elements and resistomes; and Table S5: Table showing the colony forming unit (CFU) of faecal contamination indicator bacteria and pathogenic bacteria in soil/sediment samples collected during flood (August 2018); Table S6: Table showing the geographical details of sampling sites and its environmental indices during the flood (August 2018) in Kuttanad, Kerala, India.
