# Supplementary material for: Insights into the Bacterial Profiles and Resistome Structures Following the Severe 2018 Flood in Kerala, South India
Source: Microorganisms. 2019 Oct 19;7(10):474. doi: 10.3390/microorganisms7100474 (PMC6843399; doi:10.3390/microorganisms7100474)
Supplement: Supplementary file 1 [file microorganisms-07-00474-s001.zip › Supplementary Information/Supplementary Figures.pdf]

Supplementary Figures

Insights into the bacterial profiles and resistome structures following severe 2018 flood in Kerala, South India

Soumya Jaya Divakaran [$1], Jamiema Sara Philip[$1], Padma Chereddy[1], Sai Ravi Chandra Nori[1], Akshay Jaya Ganesh[1], Jiffy John[1], Shijulal Nelson-Sathi[1*]

[1]Interdisciplinary Biology, Rajiv Gandhi Centre for Biotechnology (RGCB), Thiruvananthapuram, India

* Correspondence: *shijulalns@rgcb.res.in;* Shijulal Nelson-Sathi, Interdisciplinary Biology, Rajiv Gandhi Centre for Biotechnology (RGCB), Thiruvananthapuram, India, Phone: +91-4712781236

$ These authors contributed equally to this work

Keywords: metagenomics; flood; antibiotic resistance; bacterial diversity

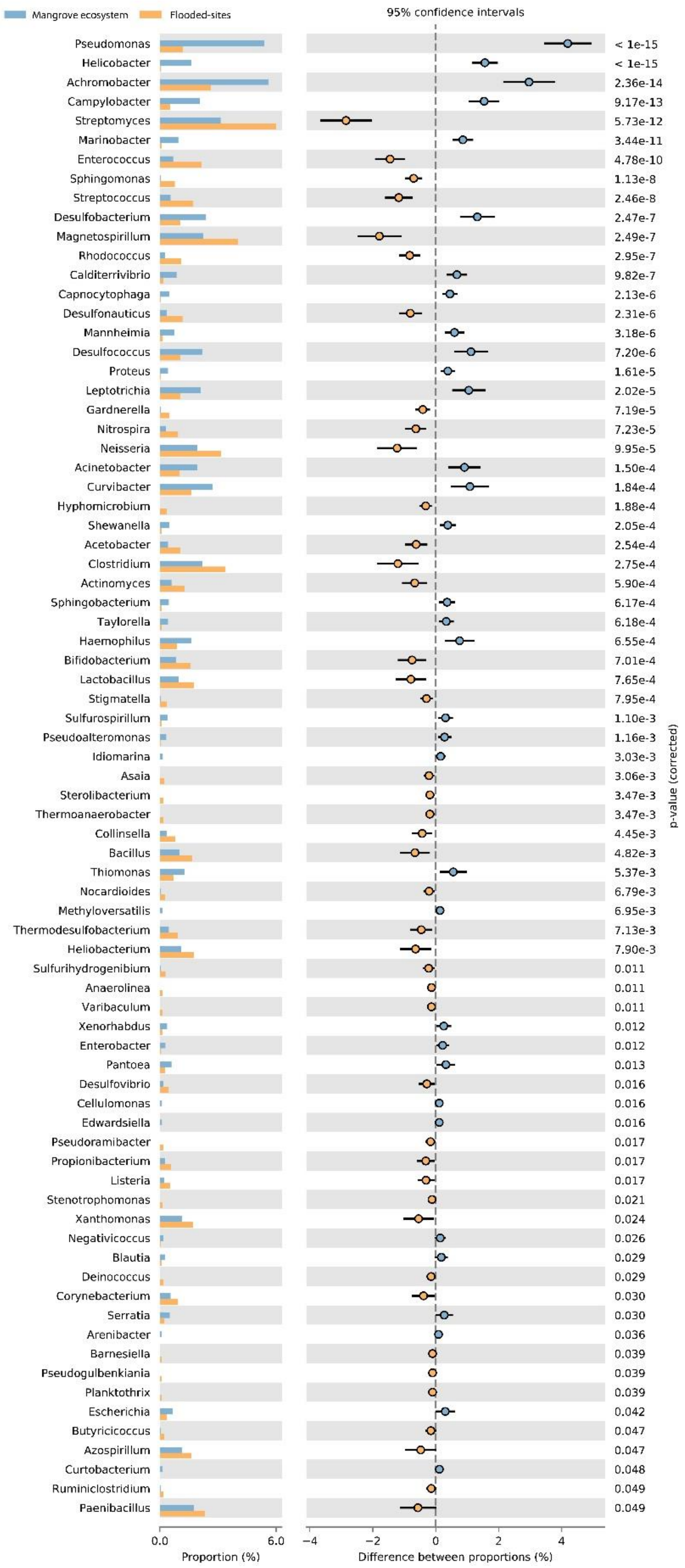

Figure S1: Distribution of 77 genera found at significantly different abundances in the metagenomics profiles of flooded and mangrove settings as identified by STAMP analysis.

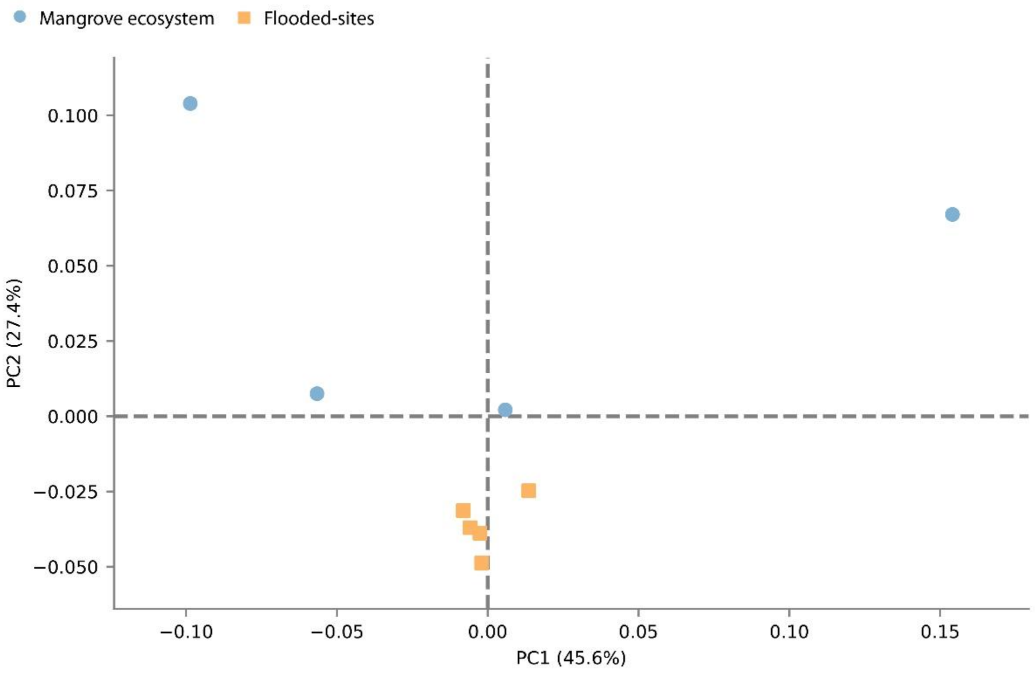

Figure S2: Principal component analysis (PCA) based on relative abundance of bacterial taxa at genus level in mangrove (blue) and flooded (orange) sites using Welch's t-test two sided ($p<0.05$).

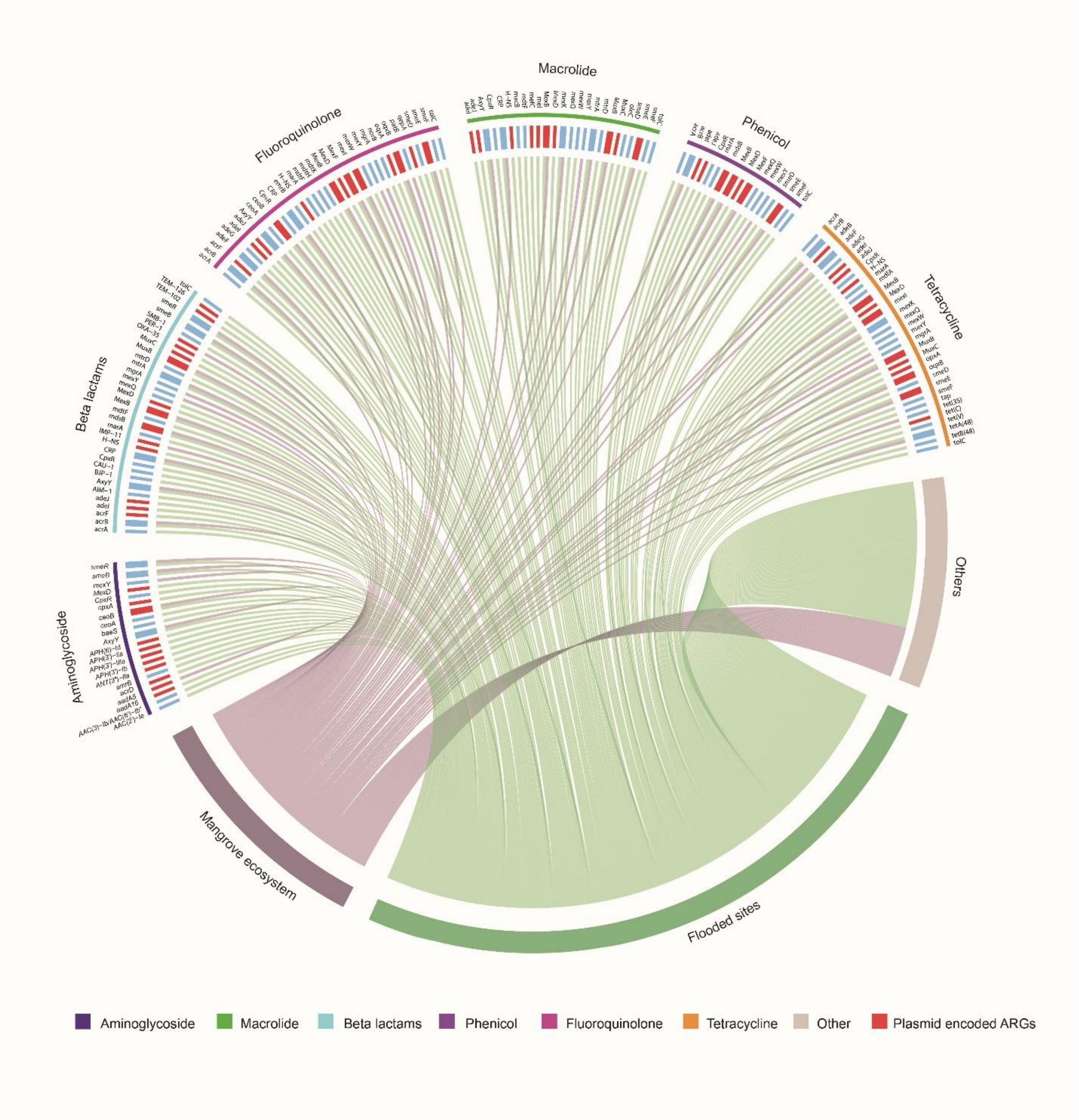

Figure S3: Chord diagram showing the presence of Antibiotic Resistant Genes (ARG) detected in flooded (green) and mangrove (grey) settings.
