# Supplementary material for: Insights into the Bacterial Profiles and Resistome Structures Following the Severe 2018 Flood in Kerala, South India
Source: Microorganisms. 2019 Oct 19;7(10):474. doi: 10.3390/microorganisms7100474 (PMC6843399; doi:10.3390/microorganisms7100474)
Supplement: Supplementary file 1 [file microorganisms-07-00474-s001.zip › Supplementary Information/Table S3.docx]

| Sample ID | Shannon diversity index, H’ | Chao1 richness |
| --- | --- | --- |
| Flooded sites | | |
| RGCB_1027 | 6.13 | 188 |
| RGCB_1028 | 6.23 | 278 |
| RGCB_1029 | 6.19 | 169 |
| RGCB_1030 | 6.54 | 271 |
| RGCB_1031 | 6.27 | 172 |
|  |  |  |

Table S3: Table showing the biodiversity indices of bacterial communities in flooded sites.
