# Supplementary material for: Insights into the Bacterial Profiles and Resistome Structures Following the Severe 2018 Flood in Kerala, South India
Source: Microorganisms. 2019 Oct 19;7(10):474. doi: 10.3390/microorganisms7100474 (PMC6843399; doi:10.3390/microorganisms7100474)
Supplement: Supplementary file 1 [file microorganisms-07-00474-s001.zip › Supplementary Information/Table S6.docx]

Table S6: Table showing the colony forming unit (CFU) of faecal indicator bacteria and pathogenic bacteria in soil/sediment samples collected during the flood (August 2018). Abundance of *Escherichia coli*, *Enterococcus faecalis*, *Vibrio chloerae*, *Klebsiella pneumoniae*, *Staphylococcus aureus*, *Pseudomonas aeruginosa* and *Salmonella* Typhi/Typhimurium are represented in CFU/gram of dry weight*.*

| Microorganism | (CFU / gram of dry weight) |
| --- | --- |
| *Staphylococcus aureus* (10^5^) | 8.5±3.0 |
| *Enterococcus faecalis* (10^3^) | 8.4±0.5 |
| *Escherichia coli* (10^3^) | 3±0.35 |
| *Salmonella* Typhi/Typhimurium (10^3^) | 12.03±4 |
| *Vibrio cholera* (10^3^) | 2.5±1 |
| *Klebsiella pneumoniae* (10^3^) | 1.8±1.36 |
| *Pseudomonas aeruginosa* (10^2^) | 3.3±1.44 |

All data are mean of triplicates; ±standard deviation (s.d).
