# Supplementary figures and images for: Insights into the Bacterial Profiles and Resistome Structures Following the Severe 2018 Flood in Kerala, South India
Source: Microorganisms. 2019 Oct 19;7(10):474. doi: 10.3390/microorganisms7100474 (PMC6843399; doi:10.3390/microorganisms7100474)

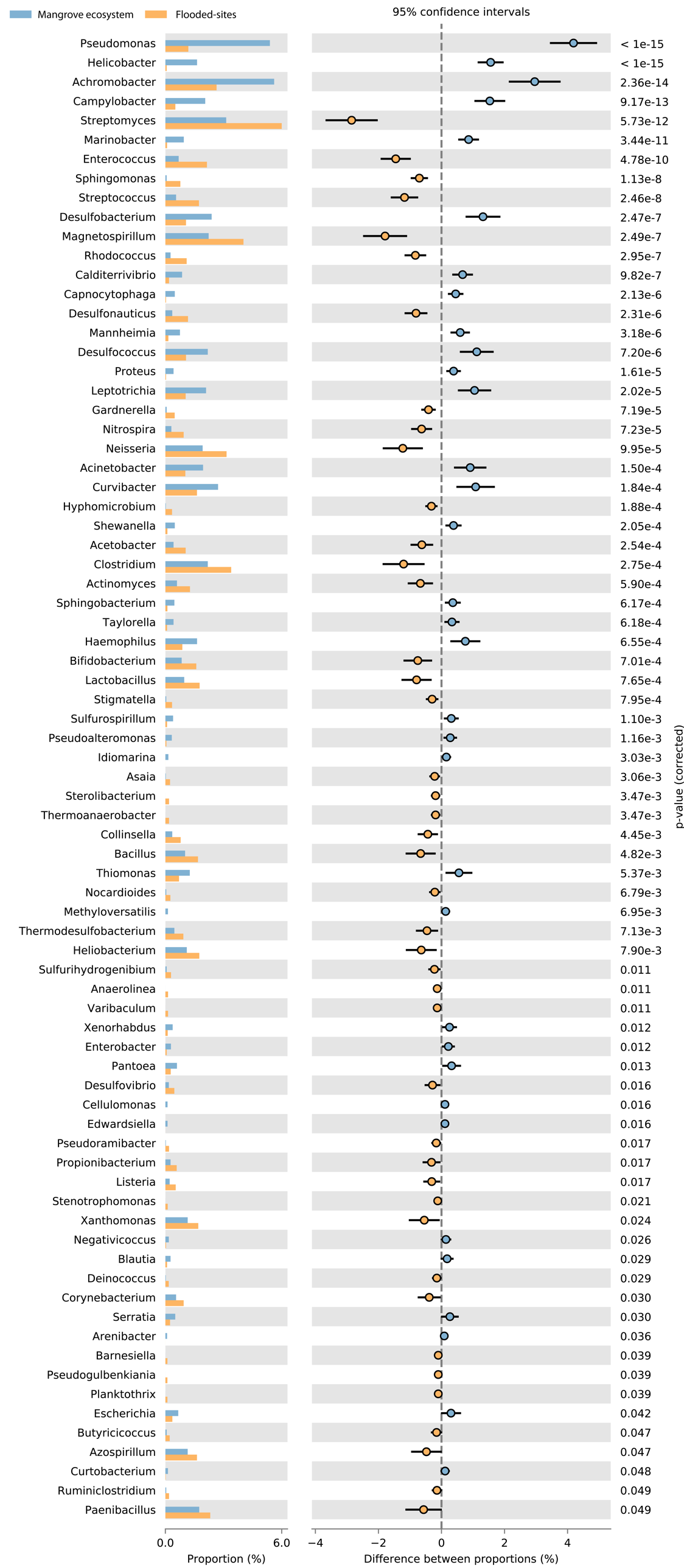

Supplement: Supplementary file 1 [file microorganisms-07-00474-s001.zip › Supplementary Information/Figure S1.jpg]

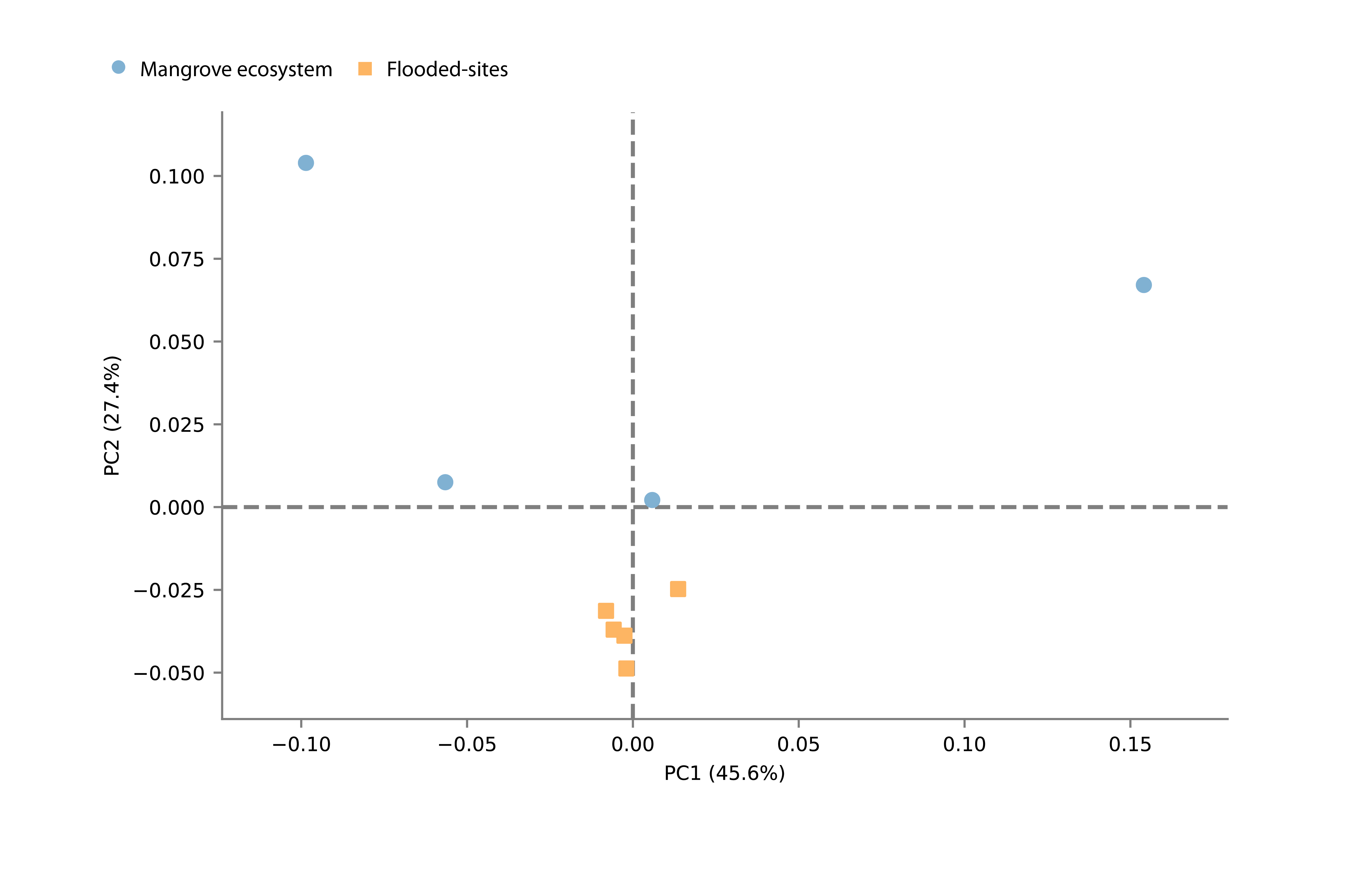

Supplement: Supplementary file 1 [file microorganisms-07-00474-s001.zip › Supplementary Information/Figure S2.jpg]

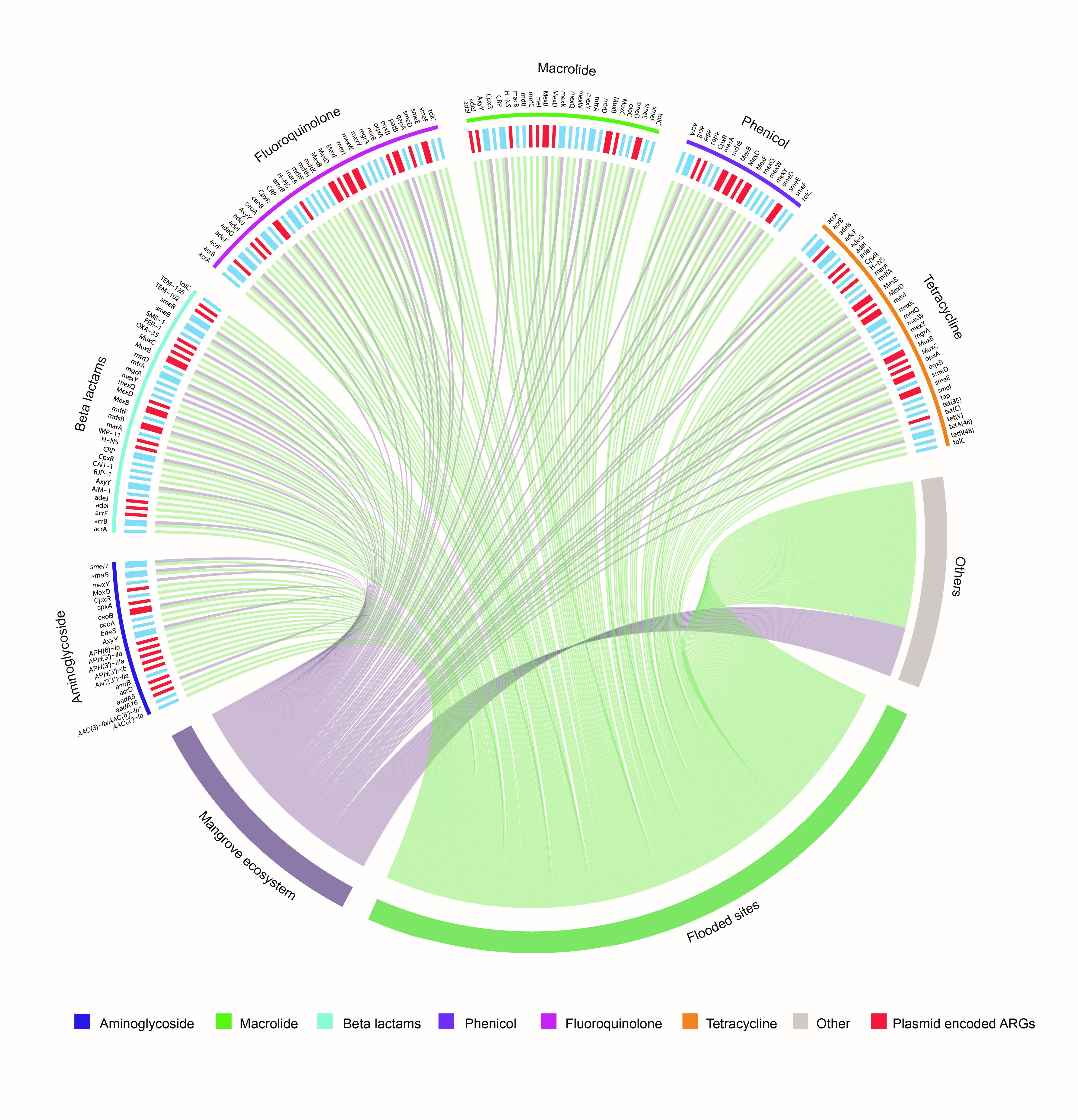

Supplement: Supplementary file 1 [file microorganisms-07-00474-s001.zip › Supplementary Information/Figure S3.jpg]
